# A Nonlinearity Mitigation Method for a Broadband RF Front-End in a Sensor Based on Best Delay Searching

**DOI:** 10.3390/s17102233

**Published:** 2017-09-28

**Authors:** Wen Zhao, Hong Ma, Hua Zhang, Jiang Jin, Gang Dai, Lin Hu

**Affiliations:** 1 School of Electronic Information and Communications, Huazhong University of Science & Technology, 1037 Luoyu Road, Wuhan 430074, China; D201177667@hust.edu.cn (W.Z.); mahong@hust.edu.cn (H.M.); jinjiang@hust.edu.cn (J.J.); daigang99@126.com (G.D.); D201077465@hust.edu.cn (L.H.); 2 Hubei Province Radio Monitoring Station, 15 Fayuan Road, Wuhan 430061, China; daigang99@126.com

**Keywords:** broadband RF front-end, nonlinear distortion, non-constant group delay distortion, nonlinearity mitigation, cognitive radio wireless sensor network

## Abstract

The cognitive radio wireless sensor network (CR-WSN) is experiencing more and more attention for its capacity to automatically extract broadband instantaneous radio environment information. Obtaining sufficient linearity and spurious-free dynamic range (SFDR) is a significant premise of guaranteeing sensing performance which, however, usually suffers from the nonlinear distortion coming from the broadband radio frequency (RF) front-end in the sensor node. Moreover, unlike other existing methods, the joint effect of non-constant group delay distortion and nonlinear distortion is discussed, and its corresponding solution is provided in this paper. After that, the nonlinearity mitigation architecture based on best delay searching is proposed. Finally, verification experiments, both on simulation signals and signals from real-world measurement, are conducted and discussed. The achieved results demonstrate that with best delay searching, nonlinear distortion can be alleviated significantly and, in this way, spectrum sensing performance is more reliable and accurate.

## 1. Introduction

Being capable of gathering information from the environment with simple processing, low complexity, and low power consumption, wireless sensor networks (WSN) have attracted an increasing amount of attention and have seen rapid development, especially in micro-electro-mechanical systems (MEMS) technology [1]. Generally, since the Industrial Scientific Medical (ISM) band, where WSNs operate, is shared with various kinds of successful communication technologies [2], the detection performance in a complex electro-magnetic environment is affected by false alarms or missed detection, and inevitably degrades. Instead of the traditional fixed-spectrum arrangement method, cognitive radio (CR) has the ability to sense underutilized or unutilized bands [3] to increase the spectrum efficiency to some extent. Hence, CR-WSN is supposed to be a promising technique, taking into account both spectrum sensing accuracy and efficiency [4,5,6].

At present, most studies regarding CR-WSN in published papers focus on the development of sensing techniques [7,8,9,10,11,12,13]. The literature [7] provides a spectrum-sensing technique based on the autocorrelation of received samples and decides whether signals exist by means of a derived Euclidean distance. In Reference [8], a spectrum sensor scheduling algorithm and a data sensor resource allocation algorithm are proposed for a heterogeneous cognitive radio sensor network (HCRSN) to achieve the sustainability of spectrum sensors and the conservation of data sensors. Except for the development on improving spectrum sensing performance, the sensing performance evaluation of CR networks is studied in Reference [9].

By contrast, the limitation of radios, as another essential reason causing vacant spectrum inaccessibility and deteriorated sensing performance, should be discussed further. Normally, the broadband RF front-end of the sensor in CRs offers the secondary users (SUs) [14] unoccupied spectrum opportunistically and, in the meantime, protects licensed primary users (PUs) from interference. If there is vacant spectrum available for SUs during a certain period, the sub-band of interest can be used for signal reception by a flexible change of the filter in the digital domain. However, strong signals from PUs in adjacent channels may generate unwanted intermodulation to interfere with SUs’ signals or occupy vacant spectrum. In this case, detection mechanisms in a CR-WSN are very likely to make the wrong judgment on the service condition of the spectrum, resulting in spectrum efficiency degradation. Therefore, for the sake of facilitating the development of CR-WSN, a broadband RF front-end with sufficient spurious-free dynamic range (SFDR) is an important guarantee.

Distortions, namely the RF front-end impairments, contain nonlinear distortion, in-phase/quadrature (I/Q) imbalance, in-band group delay distortion, carrier frequency offset, and phase noise [15]. I/Q imbalance usually takes place at the direct-conversion receiver (DCR) whose I/Q mixer fails to provide an exactly 90-degree phase shift as desired. This can be avoided through digital down-conversion (DDC) after the analog to digital converter (ADC). Additionally, de-noise methods [16,17] have been widely studied and have achieved a certain degree of noise reduction effect.

Loosely speaking, compared with other kinds of distortions in the RF front-end, nonlinear distortion is more difficult to mitigate [18], especially the spectral hyperplasia generated by strong signals inside or outside the sub-band of interest which occupies free bandwidth or hides weaker target signals away. In Reference [19], circuits were designed to receive the I/Q signal and calculate its different kinds of nonlinear distortions to compensate for the distorted baseband signals. In Reference [20], intermodulation products were generated by a tunable nonlinear circuit and depressed nonlinearities in the original signals after adaptive filtering.

In addition to the above nonlinearity suppression methods by analog circuits, a great many methods [21,22,23,24,25,26,27] based on more flexible digital signal processing (DSP) have been published. In Reference [21], a modified post-distortion algorithm was proposed to mitigate nonlinearities in a broadband receiver which is far away from the original fundamental waves, as well as to corrupt the other sub-channels. This method succeeded in designing a broadband direct digitization RF receiver. However, it could hardly adapt to compensating intermodulation products since its signal extraction is unsuitable for the components inside or nearby the adjacent channel of strong signals. In Reference [22], a nonlinear behavior model of a baseband signal was designed to eliminate nonlinearities in an RF receiver. The objective signal was converted to a baseband and then centered at DC after rotation. Then, each distortion component and its frequency content function were calculated to form an output of a nonlinear model for compensation. Unfortunately, this method has an unwanted limitation in the number of input signals. In References [23,24], a finite impulse response (FIR) band-pass/band-stop filter was formed according to the strong signals’ frequency location and practical adaptive filtering-based learning algorithms were then proposed. Our previous work [27] suggested a blind identification criterion for minimizing the short-time energy of the nonlinear components in the frequency domain. However, suffering from the non-ideal frequency response characteristic of FIR, the methods mentioned above may hardly adapt to the situation where multiple unknown signals with different power levels exist simultaneously. Unfortunately, in most cases, this is exactly the electromagnetic environment in which the RF front-end of the sensor works.

On the other hand, in-band group delay distortion, which introduces inter-symbol interference to the output signal, inevitably degrades the overall CR-WSN system performance. Many references [28,29,30,31,32,33] have been presented up to now. Due to an analog circuit’s lack of flexibility and applicability, strategies in DSP have become a trend. In Reference [34], the cumulative distribution function (CDF) of a given modulation signal was calculated and a measure of distortion of the in-band group delay was then constructed in the statistical domain, which could be optimized to obtain the equalizing filter coefficients. Nevertheless, this method cannot be utilized in the RF front-end in the sensor because the statistical characteristic of various modulation mode signals with different levels is hardly determined and, furthermore, the processing procedure of the proposed algorithm is too complicated to meet the requirement in the context of CRs.

In addition, it is worth noting that even though non-constant group delay belongs to linear distortion, when it joins with the memory nonlinear effect, the system modeling of group delay compensation and nonlinearity mitigation in series cannot achieve the desired improvement any longer. Even worse, the equalizer could increase the memory depth of the nonlinear behavior model. According to the best knowledge of the author, in the existing state-of-the-art literature, few methods involving nonlinearity mitigation take group delay distortion compensation into account.

In this paper, a simplified RF front-end model containing a low noise amplifier (LNA) and filter with a non-constant group delay in the passband is chosen as the object of analysis. Then, we emphasize how the group delay distortion brings a phase change to each frequency component at the output. Furthermore, in terms of the time domain, it can be interpreted as a delay between the strong signals producing nonlinearities and the weak signals including the nonlinear components and low signal-to-noise ratio (SNR) signals. Thereupon, the instantaneous power residual error can be regarded as a function of nonlinear model kernels and delay. Consequently, a blind identification criterion for nonlinearity mitigation with best delay searching is designed.

To eliminate the disadvantage of filter imperfections and be able to deal with multicarrier situations without too many extra operations added, strong signals and nonlinearities are extracted based on the threshold power in the frequency domain. Afterwards, their time domain waveforms are obtained by the inverse fast Fourier transform (IFFT) technique. Compared to the signal extraction realized by pass/stop-band filters, the proposed method can save not only the amplitudes of the original signals, but also their phase spectrum. This is a significant preparation step for making a reliable and precise best delay search. After that, an iterative formula based on the weighed iterative improvement (WII) [35] method is applied to minimize the ill-conditioned equation problem.

The outline of the remainder of this paper is as follows: Section 2 explains the joint effect of group delay distortion and nonlinear distortion through a simplified model from the broadband RF front-end and, based on it, the blind identification criterion is designed. Then the mitigation architecture and optimization solution are suggested in Section 3. In Section 4, simulation experiments and RF measurement results are provided to verify the performance of the proposed method. Finally, conclusions are drawn in Section 5.

## 2. System Model and Problem Analysis

As mentioned above, the broadband RF front-end with sufficient linearity is an important guarantee of a reliable spectrum sensing. In this section, first, a classical broadband RF front-end of a sensor is chosen as an example and then a simplified model is built to discuss its joint effect of group delay distortion and nonlinear distortion. Finally, an identification criterion for nonlinearity mitigation is suggested. 

### 2.1. RF Front-End of a Sensor Node in CR-WSN

**Figure 1:**
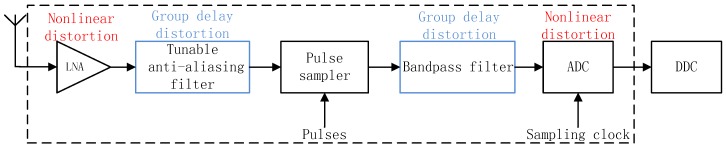
Broadband reconfigurable direct RF front-end.

A broadband reconfigurable direct RF front-end architecture is shown in Figure 1. The input signals from the antenna are amplified by the LNA. After that, a tunable anti-aliasing filter configures the center frequency to determine the frequency location to be sampled. Then, by a series of pulses, the pulse sampler down-converts the signals to a certain intermediate frequency (IF). The second band-pass filter removes the components outside the IF bandwidth so that signals can be sampled and converted to digital signals by ADC. Finally, baseband signals are available through DDC. 

It is worth noting that, except for the heterodyne receiver shown in Figure 1, zero-IF and low-IF receivers are also in general use in sensors. However, no matter which kind the receiver belongs to, the joint effect of the non-constant group delay distortion and nonlinear distortion could occur in the receiver. Strictly speaking, an ideal linearity condition is hardly met in a real RF front-end. Almost every part in a receiver, such as the LNA, mixer, BPF, and ADC, can generate nonlinear components. Moreover, as long as the RF front-end has a broad passband, group delay is supposed to vary in a large range that cannot be ignored and, especially, the BPF’s nonlinear phase-frequency response and the nonlinear device’s memory effect will both make it happen. Thus, in the actual receiver system, the joint effect certainly exists and becomes more complicated. 

Besides these two kinds of distortions, in zero-IF or low-IF receivers, another significant effect resulting in decreased SFDR is I/Q imbalance, which is due to an imprecise 90-degree phase shift in the I/Q mixer. The main objective of this paper is to analyze the joint effect of non-constant group delay distortion and nonlinear distortion of a broadband RF front-end and provide its corresponding solution. In order to highlight the key point and make the result of the proposed method simple and explicit, a heterodyne receiver is taken as the object of study.

### 2.2. Joint Effect of Group Delay Distortion and Nonlinear Distortion

**Figure 2:**
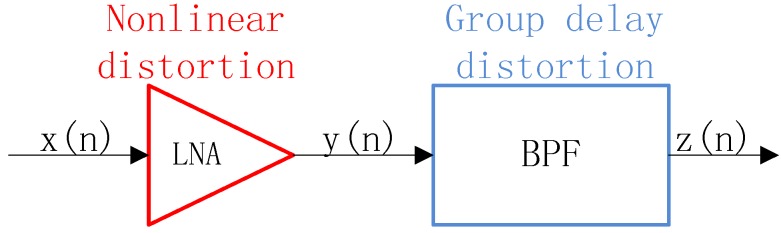
Simplified model.

According to Figure 1, the proposed method is expected to focus on baseband signals after the RF front-end and DDC. In order to see how a non-constant group delay in the passband influences the signals with nonlinearity, a simplified grey-box model containing the LNA and band-pass filter (BPF) in series is built in Figure 2. When passing through the receive chain, signals are distorted. Even though all kinds of modules with non-ideal linear characteristics in the RF front-end could bring in nonlinearity and non-constant group delay, we should pay attention to the main influence and make the following assumptions that the main source where nonlinearities are generated is at the LNA stage, and non-constant group delay distortion in the passband is due to the non-ideal phase-frequency response of the BPF.

Let x(n) be the chosen signals with a fixed center frequency and bandwidth. After the LNA, y(n) are signals containing nonlinearity. z(n) means the signals passing through the BPF are distorted both by nonlinear products and non-constant group delay distortion. If no BPF exists, our previous method [27] presents a blind identification criterion of forming a nonlinear behavior model to compensate for nonlinear interference:

(1) $$s_{y}(n)=y(n)-f\{y(n)\}$$

where $s_{y}(n)$ are the compensated signals and f{⋅} indicates the nonlinear behavior model, such as the Volterra model and Hammerstein model. 

Generally speaking, a constant group delay in the passband of RF filter circuits is desirable. However, it is difficult to achieve in broadband systems, or even in narrowband communications, when tight filtering is required [15]. Since the hypothesis is that the BPF only brings the non-constant group delay to y(n) , it can be regarded as a linear filter adding nonlinear phase increments to each frequency component in the passband. From the perspective of the frequency domain, to each frequency spectrum:

(2) $$Z(k)=Y(k)\cdot H(k)=Y(k)\cdot A(k)e^{j\varphi (k)}$$

where Y(k) and Z(k) are the frequency spectra of y(n) and z(n) , respectively. Their frequency value is represented as k (k=0,1,2,...,N−1) . N is the number of points carrying out the discrete Fourier transform (DFT). H(k) represents the frequency response function of the BPF whose amplitude is A(k) and phase is φ(k) . Then, the Inverse Discrete Fourier Transform (IDFT) of Z(k) is expressed as:

(3) $$z(r)=\frac{1}{N}\sum_{k=0}^{N-1}Z(k)e^{j\frac{2\pi }{N}kr}$$

and the DFT of y(n) is:

(4) $$Y(k)=\sum_{n=0}^{N-1}y(n)e^{-j\frac{2\pi }{N}kn}$$

Substituting Equations (2) and (4) into Equation (3):

(5) $$\begin{array}{ll} z(r) & =\frac{1}{N}\sum_{k=0}^{N-1}Y(k)A(k)e^{j\varphi (k)}e^{j\frac{2\pi }{N}kr}\\ & =\frac{1}{N}\sum_{k=0}^{N-1}A(k)e^{j\varphi (k)}\sum_{n=0}^{N-1}y(n)e^{-j\frac{2\pi }{N}kn}e^{j\frac{2\pi }{N}kr}\\ & =\frac{1}{N}\sum_{n=0}^{N-1}y(n)\sum_{k=0}^{N-1}A(k)e^{j[\frac{2\pi }{N}k(r-n)+\varphi (k)]} \end{array}$$

In Reference [34], the phase characteristic of the BPF is modeled by a *P*th order polynomial function and, without loss of generality, we obtain:

(6) $$\varphi (k)=\frac{2\pi }{N}\sum_{p=1}^{P}c_{p}k^{p}$$

Next, we employ FIR to simulate which has the same amplitude response and phase response as the BPF in Figure 2. Since the phase response of the BPF is nonlinear in the passband, there exists no linear relationship between φ(k) and k . Meanwhile, considering that the time domain response coefficients of FIR belong to the real number field, to meet to the symmetry condition, we suppose that A(k) has even symmetry and φ(k) has odd symmetry relative to $k=\frac{N}{2}$ ( N is an even number), i.e., A(k)=A(N−k) , φ(k)=−φ(N−k) , and $A(\frac{N}{2})=0$ , $\varphi (\frac{N}{2})=0$ . Then, Equation (5) can be written as:

(7) $$\begin{array}{ll} z(r) & =\frac{1}{N}\sum_{n=0}^{N-1}y(n)\sum_{k=0}^{N-1}A(k)e^{j[\frac{2\pi }{N}k(r-n)+\varphi (k)]}\\ & =\frac{1}{N}\sum_{n=0}^{N-1}y(n)\{A(0)+\sum_{k=1}^{\frac{N}{2}-1}A(k)e^{j[\frac{2\pi }{N}k(r-n)+\varphi (k)]}+\sum_{k=\frac{N}{2}+1}^{N-1}A(k)e^{j[\frac{2\pi }{N}k(r-n)+\varphi (k)]}\}\\ & =\frac{1}{N}\sum_{n=0}^{N-1}y(n)\{A(0)+\sum_{k=1}^{\frac{N}{2}-1}A(k)e^{j[\frac{2\pi }{N}k(r-n)+\varphi (k)]}+\sum_{k^{'}=\frac{N}{2}-1}^{1}A(N-k^{'})e^{j[\frac{2\pi }{N}(N-k^{'})(r-n)+\varphi (N-k^{'})]}\}\\ & =\frac{1}{N}\sum_{n=0}^{N-1}y(n)\{A(0)+\sum_{k=1}^{\frac{N}{2}-1}A(k)e^{j[\frac{2\pi }{N}k(r-n)+\varphi (k)]}+\sum_{k^{'}=1}^{\frac{N}{2}-1}A(k^{'})e^{-j[\frac{2\pi }{N}k^{'}(r-n)+\varphi (k^{'})]}\} \end{array}$$

Apparently, $\varphi (0)=\sum_{p=1}^{P}c_{p}0^{p}=0$ . Utilizing the transformation $k=N-k^{'}$ , and since k and $k^{'}$ are operated in the same accumulation range, Equation (7) can be written as:

(8) $$z(r)=\frac{1}{N}\sum_{n=0}^{N-1}y(n)\{A(0)+2\sum_{k=1}^{\frac{N}{2}-1}A(k)\cos[\frac{2\pi }{N}k(r-n)+\varphi (k)]\}$$

If the BPF has a linear phase response, $\varphi_{ideal}(k)=\frac{2\pi }{N}c_{1}k$ , the second term in Equation (8) becomes:

(9) $$\sum_{k=1}^{\frac{N}{2}-1}A(k)\cos[\frac{2\pi }{N}k(r-n)+\varphi (k)]=\sum_{k=1}^{\frac{N}{2}-1}A(k)\cos[\frac{2\pi }{N}k(r-n+c_{1})]$$

This means that for a fixed time r , there exists $n=r+c_{1}$ making:

(10) $$\sum_{k=1}^{\frac{N}{2}-1}A(k)\cos[\frac{2\pi }{N}k(r-n+c_{1})]=\sum_{k=1}^{\frac{N}{2}-1}A(k)$$

While $n\neq r+c_{1}$ , the odd symmetry of the cosine function in the range of 0 to π makes this accumulation term equal to 0. Hence, we obtain:

(11) $$z(r)=\frac{1}{N}A(0)\sum_{n=0}^{N-1}y(n)+\frac{2}{N}\sum_{k=1}^{\frac{N}{2}-1}A(k)y(n-c_{1})$$

On account of $A(0)<<\sum_{k=1}^{\frac{N}{2}-1}A(k)$ and the direct component having no variation in phase, the final expression is:

(12) $$z(r)=\frac{2}{N}y(n-c_{1})\sum_{k=1}^{\frac{N}{2}-1}A(k)$$

Equation (12) means that each frequency component in y(n) has an equal delay $c_{1}$ when passing by the ideal BPF and they are outputted at the same time.

However, if the phase variation φ(k) relative to frequency k is nonlinear, the manner that y(n) passes through the BPF is similar to the scattering of electromagnetic wave propagation. To be specific, $Y(k_{1})$ will be outputted by the BPF after delay $\tau_{k_{1}}$ which is different from $\tau_{k_{2}}$ , the delay of $Y(k_{2})$ , because $\tau_{k}$ is related to a polynomial function of frequency k . In this way, nonlinear products outputted at the same time n in y(n) are sampled at different moments of z . Taking this effect into account when designing the nonlinearity identification criterion, modeling is expected to be more accurate and distortion will be mitigated more thoroughly. Thus:

(13) $$s_{z}(n)=z(n)-f\{z(n),\tau \}$$

To be exact, τ should be a function of frequency. However, to make the realization easy, we choose a simplified method, in that:

(14) $$s_{z}(n)=z(n-\tau_{best})-f\{z(n)\}$$

Here, $\tau_{best}$ is not a delay really existing in the RF front-end, but a sampling interval between the output of the nonlinear behavior model and the distortion component in z . When the best delay is found, the minimum of the power residuals after optimization is expected to be more precise. In addition, the searching range to which the best delay belongs can be set according to prior knowledge of the *S* parameter of the RF front-end. Once the *S* parameter is obtained, it can be determined by the differential of the phase angle of $S_{21}$ to the frequency.

### 2.3. Design of Blind Identification Criterion

**Figure 3:**
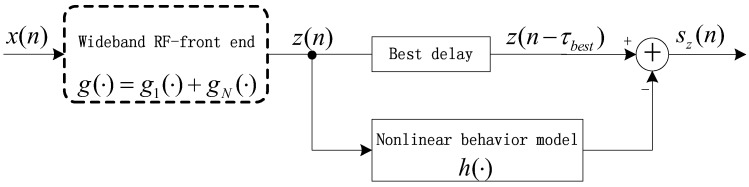
Principle of nonlinearity mitigation.

The scheme for nonlinearity cancellation is presented in Figure 3, where g(⋅) is assumed as the transfer function of a broadband RF front-end including the linear term with memory $g_{1}(\cdot )$ and the nonlinear term with memory $g_{N}(\cdot )$ . Hence:

(15) $$z(n)=g[x(n)]=g_{1}[x(n)]+g_{N}[x(n)]$$

The input signals x(n) could be divided into strong signals $x_{h}(n)$ which produce nonlinear interference when passing through the RF front-end, and weak signals $x_{l}(n)$ containing nonlinear distortions, low power signals, and noise $n_{x}(n)$ , namely:

(16) $$x(n)=x_{h}(n)+x_{l}(n)+n_{x}(n)$$

Substituting Equation (16) into (15):

(17) $$\begin{array}{ll} z(n) & =g_{1}[x_{h}(n)]+g_{1}[x_{l}(n)]+g_{N}[x_{h}(n)]+g_{N}[x_{l}(n)]+g_{N}[x_{h}(n),x_{l}(n)]+n_{z}(n)\\ & \approx g_{1}[x_{h}(n)]+g_{1}[x_{l}(n)]+g_{N}[x_{h}(n)]+n_{z}(n) \end{array}$$

Normally, as a result of $x_{h}(n)>>x_{l}(n)$ , $g_{N}[x_{l}(n)]$ and $g_{N}[x_{h}(n),x_{l}(n)]$ are much less than $g_{N}[x_{h}(n)]$ and below the noise floor; thus, they can be neglected in z(n) . If h(⋅) is the nonlinear behavior model and meets the condition that $h[g_{1}(\cdot )]=g_{N}(\cdot )$ , based on the discussion in Section 2.2, we obtain:

(18) $$\begin{array}{ll} s_{z}(n) & =z(n-\tau_{best})-h[z(n)]\\ & =g_{1}[x_{h}(n-\tau_{best})]+g_{1}[x_{l}(n-\tau_{best})]+g_{N}[x_{h}(n-\tau_{best})]+n_{z}(n-\tau_{best})\\ & \;-h\{g_{1}[x_{h}(n)]+g_{1}[x_{l}(n)]+g_{N}[x_{h}(n)]+n_{z}(n)\}\\ & \approx g_{1}[x_{h}(n-\tau_{best})]+g_{1}[x_{l}(n-\tau_{best})]+g_{N}[x_{h}(n-\tau_{best})]-h\{g_{1}[x_{h}(n)]\}+n_{s}(n)\\ & =g_{1}[x_{h}(n-\tau_{best})]+g_{1}[x_{l}(n-\tau_{best})]+n_{s}(n) \end{array}$$

Since $x_{h}(n)>>x_{l}(n)$ , there is an assumption that the output of the nonlinear behavior model $h\{g_{1}[x_{h}(n)]+g_{1}[x_{l}(n)]+g_{N}[x_{h}(n)]+n_{z}(n)\}\;\approx h\{g_{1}[x_{h}(n)]\}$ . Actually, Equation (18) provides the identification criterion of nonlinear mitigation. To each delay within the searching range, optimization can be employed to make the power residual between $g_{N}[x_{h}(n-\tau_{best})]$ and $h\{g_{1}[x_{h}(n)]\}$ achieve its minimal value. The minimal one among these values is acquired at the best delay and, hence, the corresponding coefficients of the nonlinear model are regarded as the optimal solution for nonlinearity mitigation.

## 3. Proposed Mitigation Architecture for an RF Front-End

Based on the identification criterion above, in this section, mitigation architecture is proposed in detail and followed by WII optimization method for nonlinear model kernels calculation.

### 3.1. Mitigation Architecture

**Figure 4:**
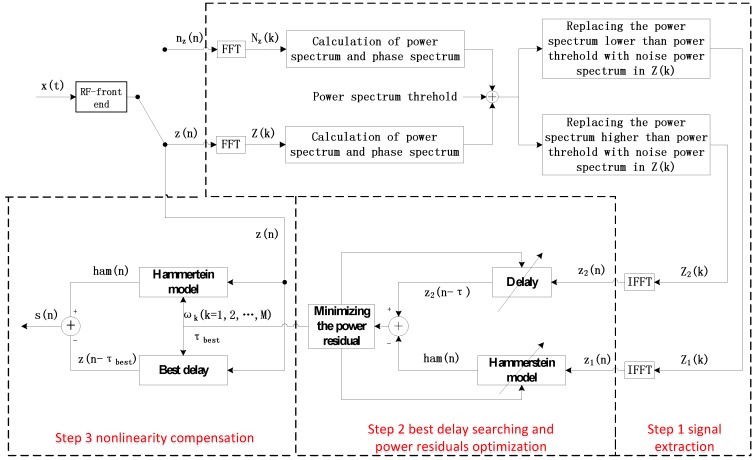
Mitigation architecture.

As shown in Figure 4, mitigation architecture can be divided into three steps including signal extraction, best delay searching and power residuals optimization, and nonlinearity compensation. Since the reason and method of step 3 have been introduced in Section 2, step 1 and step 2 will be discussed in this section. 

Let us begin with the process of step 1. Signals x(t) are first sampled and converted to digital baseband signals z(n) . Subsequently, noise $n_{z}(n)$ is obtained when the RF front-end is at the same working state of receiving z(n) , such as the center frequency, passband bandwidth, system gain, and attenuation. Then, a fast Fourier transform (FFT) is carried out to obtain the frequency spectra of z(n) and $n_{z}(n)$ . By substituting the power spectrum of z(n) for the noise power spectrum according to the set threshold, strong signals $Z_{1}(k)$ and weak signals $Z_{2}(k)$ in the frequency domain are separated from z(n) , respectively. Finally, the waveforms of $z_{1}(n)$ and $z_{2}(n)$ in the time domain are available through IFFT. 

It is worth mentioning that this type of signal extraction saves both amplitudes and phases of the original signal and, hence, has the following benefits. In the literature [23,27], FIRs are utilized. Suffering from imperfect performance of amplitude-frequency and phase-frequency response, there will be unwanted amplitude and phase changes in $z_{1}(n)$ and $z_{2}(n)$ . This is inevitably harmful for best delay searching and nonlinear behavior model kernel calculation. In addition, compared with continually designing and changing multi-stopband/multi-passband digital filters with extremely high performance, in the proposed approach, only power threshold altering is needed. Therefore, this method is more likely to handle the situation of multiple signals with different power levels or wide ranges of bandwidth, which is apparently more flexible and convenient. 

In the rest of Section 3, step 2 is explained in detail including the choice of nonlinear behavior model for complex baseband signal in Section 3.1, the objective function establishment in Section 3.1, and the optimization solution in Section 3.2.

The generalized Hammerstein model [24] is chosen to simulate the nonlinear behavior in the RF front-end. If the input is x(n) , the expression is:

(19) $$ham(n)=\omega_{1}(n)*x(n)+\omega_{2}(n)*x^{2}(n)+\omega_{3}(n)*x^{3}(n)+...$$

where $\left\{\omega_{1}(n),\omega_{2}(n),\omega_{3}(n)\right\}$ are impulse responses taking memory effect into account for each order, and ham(n) is the output. Even-order nonlinear components are usually far away from others [18] in the spectrum, and even if the bandwidth of the RF front-end is extremely wide, proper circuit design methodologies [18,36] can provide a high second-order intercept point where harmful effects resulting from the even-order nonlinearity are kept to a minimum. On the contrary, the odd-order nonlinearity appearing at the frequency near or inside the total frequency band of interest is hardly eliminated simply by some analog filters and, hence, more complicated to deal with. In the baseband, most nonlinearities belong to odd-order intermodulation components, and the third-order and fifth-order spectrum hyperplasia are much stronger than others. Therefore, Equation (19) is simplified to:

(20) $$ham(n)=\omega_{1}(n)*z_{1}(n)+\omega_{3}(n)*{\left|z_{1}(n)\right|}^{2}z_{1}(n)+\omega_{5}(n)*{\left|z_{1}(n)\right|}^{4}z_{1}(n)$$

In Equation (20), "|⋅|" represents the modulus operator, as $z_{1}(n)$ is the baseband complex signal and notation "∗" denotes convolution. Let $M_{1}$ , $M_{3}$ , and $M_{5}$ be the memory depth of each order. The total number of kernels is $M=M_{1}+M_{3}+M_{5}$ . Equation (20) can be expressed in a matrix form:

(21) $$ham(n)=\omega^{T}u(n)$$

where:

(22) $$\omega ={\left[\begin{array}{ccc} \begin{array}{cccc} \omega_{0,1} & \omega_{1,1} & ... & \omega_{M_{1}-1,1} \end{array} & \begin{array}{cccc} \omega_{0,3} & \omega_{1,3} & ... & \omega_{M_{3}-1,3} \end{array} & \begin{array}{cccc} \omega_{0,5} & \omega_{1,5} & ... & \omega_{M_{5}-1,5} \end{array} \end{array}\right]}^{T}$$

and:

(23) $$\begin{array}{ll} u(n)= & [\begin{array}{cccc} z_{1}(n) & z_{1}(n-1) & ... & z_{1}(n-M_{1}+1) \end{array}\\ & \begin{array}{cccc} {\left|z_{1}(n)\right|}^{2}z_{1}(n) & {\left|z_{1}(n-1)\right|}^{2}z_{1}(n-1) & ... & {\left|z_{1}(n-M_{3}+1)\right|}^{2}z_{1}(n-M_{3}+1) \end{array}\\ & \begin{array}{cccc} {\left|z_{1}(n)\right|}^{4}z_{1}(n) & {\left|z_{1}(n-1)\right|}^{4}z_{1}(n-1) & ... & {\left|z_{1}(n-M_{5}+1)\right|}^{4}z_{1}(n-M_{5}+1) \end{array}{]}^{T} \end{array}$$

The next step is to calculate the error signals between the outputs of the Hammerstein model ham(n) and the delayed weak signal $z_{2}(n-\tau )$ , while the power residual E(ω,τ) is regarded as the objective function to be minimized. Thus, E(ω,τ) is:

(24) $$E(\omega ,\tau )=\sum_{n=1}^{N}{\left|ham(n)-z_{2}(n-\tau )\right|}^{2}={\left[U\omega -Z_{2}(\tau )\right]}^{H}[U\omega -Z_{2}(\tau )]$$

where $U^{T}=\left[\begin{array}{cccc} u(1) & u(2) & ... & u(N) \end{array}\right]$ , $Z_{2}(\tau )={\left[\begin{array}{cccc} z_{2}(1) & z_{2}(2) & ... & z_{2}(N) \end{array}\right]}^{T}$ and the notation *H* is the transposed-conjugate operator. Once the searching range of the best delay is determined, the optimization problem becomes:

(25) $$\underset{\omega ,\tau }{\arg\min}E(\omega ,\tau )$$

Equation (25) reveals that for each delay τ within the searching range, E(ω,τ) reaches its minimum value after optimization. Among these values, the minimal one is chosen as the optimum solution and its corresponding τ and ω are taken as the best delay and the coefficients of nonlinear behavior kernels. As ω in the objective function belonging to the complex number field, applying deviation directly to E(ω,τ) is infeasible. Let:

(26) ωk=ρkejφk, d=UHZ2(τ)=[d1d2...dM]T, R=UUH=[r11r12...r1Mr21r22...r2M............rM1rM2...rMM]

where $\rho_{k}$ and $\varphi_{k}$ represent the amplitude and phase of $\omega_{k}$ , respectively. From Equation (24), we obtain:

(27) $$E(\omega ,\tau )=\omega^{H}R\omega -\omega^{H}d-d^{H}\omega +Z_{2}^{H}(\tau )Z_{2}(\tau )$$

Taking the derivative of E(ω,τ) with respect to $\rho_{k}$ and $\varphi_{k}$ :

(28) $$\frac{\partial E}{\partial \rho_{k}}=e^{-j\varphi_{k}}\sum_{i=1}^{M}r_{ki}\omega_{i}+e^{j\varphi_{k}}\sum_{i=1}^{M}\omega_{i}^{*}r_{ik}-d_{k}^{*}e^{j\varphi_{k}}-e^{-j\varphi_{k}}d_{k}=0$$

(29) $$\frac{\partial E}{\partial \varphi_{k}}=-j\rho_{k}e^{-j\varphi_{k}}\sum_{i=1}^{M}r_{ki}\omega_{i}+j\rho_{k}e^{j\varphi_{k}}\sum_{i=1}^{M}\omega_{i}^{*}r_{ik}-jd_{k}^{*}\rho_{k}e^{j\varphi_{k}}+j\rho_{k}e^{-j\varphi_{k}}d_{k}=0$$

Let: $\varphi =\left[\begin{array}{llll} \;e^{j\varphi_{1}} & & & \\ & e^{j\varphi_{2}} & & \\ & & ...\\ & & & e^{j\varphi_{M}} \end{array}\right]$ , $\rho =\left[\begin{array}{llll} \;\rho_{1} & & & \\ & \rho_{2} & & \\ & & ... & \\ & & & \rho_{M} \end{array}\right]$ , $\frac{\partial E}{\partial \rho }={\left[\frac{\partial E}{\partial \rho_{1}}\;\frac{\partial E}{\partial \rho_{2}}\;...\;\frac{\partial E}{\partial \rho_{M}}\right]}^{T}$ , $\frac{\partial E}{\partial \varphi }={\left[\frac{\partial E}{\partial \varphi_{1}}\;\frac{\partial E}{\partial \varphi_{2}}\;...\;\frac{\partial E}{\partial \varphi_{M}}\right]}^{T}$ and substituting them into Equations (28) and (29):

(30) $$\frac{\partial E}{\partial \rho }=\varphi^{*}R\omega +\varphi R^{*}\omega^{*}-\varphi d^{*}-\varphi^{*}d=0$$

(31) $$\frac{\partial E}{\partial \varphi }=-j\rho \varphi^{*}R\omega +j\rho \varphi R^{*}\omega^{*}-j\rho \varphi d^{*}+j\rho \varphi^{*}d=0$$

Combining Equations (30) and (31), the result is Rω=d , i.e.,

(32) UUHω=UZH2(τ)

### 3.2. Optimization Solution

To solve linear Equation (32), whose solutions ω are a series of complex numbers, we make a transformation by assuming $R=R_{re}+jR_{im}$ , $d=d_{re}+jd_{im}$ , and $\omega =\omega_{re}+j\omega_{im}$ , where subscripts “re” and “im” mean the real and imaginary parts, respectively. Thus:

(33) $$\left[\begin{array}{ll} R_{re} & -R_{im}\\ R_{im} & R_{re} \end{array}\right]\left[\begin{array}{l} \omega_{re}\\ \omega_{im} \end{array}\right]=\left[\begin{array}{l} d_{re}\\ d_{im} \end{array}\right]$$

If $A=\left[\begin{array}{ll} R_{re} & -R_{im}\\ R_{im} & R_{re} \end{array}\right]$ , $\bar{d}={\left[d_{re}{}^{T}\;d_{im}{}^{T}\right]}^{T}$ , $\bar{\omega }={\left[\omega_{re}{}^{T}\;\omega_{im}{}^{T}\right]}^{T}$ , Equation (33) can be written as:

(34) $$A\bar{\omega }=\bar{d}$$

In Equation (32), the odd-order Hammerstein model is set from 1 to 5 so that the first diagonal element of UUH is $\sum_{n=1}^{N}{\left|z_{1}(n)\right|}^{2}$ and the last one is $\sum_{n=1}^{N}{\left|z_{1}(n-M_{5}+1)\right|}^{10}$ . As a result of too large a power difference, UUH and A easily tend to be ill-conditioned matrices. In this paper, WII [35] is used to deal with this ill-conditioned problem. The prerequisite of adopting WII is that A is a real symmetry non-negative definite matrix. Since R is a Hamiltonian matrix $R=R^{H}$ and $\omega^{H}R\omega ={\left(U\omega \right)}^{H}(U\omega )={\left|U\omega \right|}^{2}\geq 0$ , it can easily be verified. The final iterative formula is:

(35) $${\bar{\omega }}^{\left(k+1\right)}={\bar{\omega }}^{\left(k\right)}+{\left(A+\lambda I\right)}^{-1}(\bar{d}-A{\bar{\omega }}^{\left(k\right)})$$

where λ is the weighting factor. Although there is a matrix inversion step appearing in Equation (35), ${\left(A+\lambda I\right)}^{-1}$ does not update during the whole iteration procedure. Hence, unlike other methods, such as the recursive least squares method (RLS), ${\left(A+\lambda I\right)}^{-1}$ can be computed in advance and stays unchanged until the entire delay searching is finished. Therefore, the recurrence formula (Equation (35)) only contains some simple multiply-add operations, making it suitable for efficient realization in software-defined radio (SDR).

## 4. Simulation Experiments and Results Analysis

In Section 4, the proposed method is utilized to evaluate its SFDR improvement, both on simulation signals and signals from a real RF front-end. To illustrate the advantages of the best delay-based nonlinear mitigation, the spectrum after nonlinear compensation at the best delay is compared with the one without delay, and the curves of optimized power residuals E(ω,τ) in Equation (24) at each delay in the searching range are also provided. Then, constellation diagrams are obtained to verify the demodulation improvement and SFDR enhancement. Finally, by the energy detection (ED) method [37], sensing performance promotion is offered.

### 4.1. The Verification of Simulation Signals

**Figure 5:**
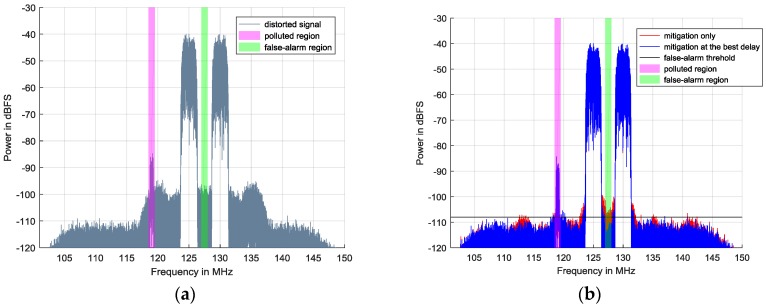
Nonlinearity mitigation results of simulation signals including two strong 16QAM signals and one weak 16QAM signal: (**a**) distorted signals; and (**b**) signals after nonlinearity mitigation.

The simulation signals are comprised of two strong 16 Quadrature Amplitude modulation (16QAM) signals and a weak one. Both of the strong signals are generated with the same amplitude of −45 dBFS and the same symbol rate of 2 MHz. Their center frequencies are 125 MHz and 130 MHz, respectively. The weak one located in the polluted region is at 119 MHz with −97 dBFS, and its symbol rate is 0.5 MHz. Their baseband signals are obtained by carrying out DDC. Hence, from the distorted spectrum in Figure 5a, unwanted nonlinearities cover the weak 16QAM’s spectrum and distort it seriously so that its signal to interference plus noise ratio (SINR) inevitably decreases. In addition, the green region affected by intermodulation is originally supposed to be a vacant spectrum. Therefore, false-alarm probability in this region rises and spectrum efficiency degrades.

**Figure 6:**
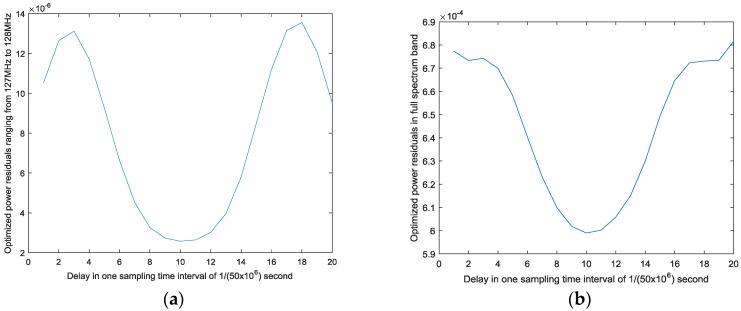
Relationships between delay and power residuals after optimization: (**a**) optimized power residuals in false-alarm region; and (**b**) optimized power residuals in full spectrum band.

Figure 6b provides the relationship between the delay and optimized power residuals in the full-spectrum band. When τ equals 10, the optimized residuals reach the minimum value compared to the others. Therefore, 10 is chosen as the best delay. In Figure 5b, the blue spectrum indicates the mitigation performance when best delay searching is employed, while the red spectrum shows the compensation result without the best delay. The nonlinear behavior model adopted here is the Hammerstein model in Equation (20) and the model orders are 1, 3, and 5 with the same memory depth of 20. By carrying out mitigation methods, SFDR in the full band rises by 10–15 dB. 

In Figure 5b, it seems that both the proposed mitigation methods have achieved satisfactory results, while the apparent differences between them are only focused on the adjacent channels of strong 16QAMs. However, these differences are crucial evidence for explaining the advantage of the best delay-based method. In Figure 5a, there mainly exist two kinds of intermodulation products. The first kind is generated when one strong 16QAM is modulated by another. In Figure 5a, they are located at the center of 120 MHz and 135 MHz and have a 9 MHz bandwidth, since the roll-off factor is 0.5. The second is caused by the strong signal itself and is located in an adjacent channel to the strong 16QAM. In the first adjacent channels of the strong 16QAMs, different kinds of distortions are vector overlapped in the frequency domain. For the first type of intermodulation product, both mitigation methods have a significant improvement while, for the second one, the best delay-based mitigation method outperforms the mitigation-only method. In particular, in the marked false-alarm region ranging from 127 MHz to 128 MHz, the spectrum after mitigation at the best delay is about −110 dBFS, which is close to the background noise level and 5 dB lower than that without the best delay. If the black line of −108 dBFS is used as the ED threshold, the red spectrum will always be detected as occupied so that SUs would miss a transmission opportunity. This is also proved by Figure 6a, which shows the relationship between the delay and optimized power residuals in the false-alarm region. The best delay of 10 corresponds to its minimal optimized power residuals. Consequently, we conclude that when the best delay is determined, the nonlinear behavior model can provide better outputs to deal with the more complicated situation where different kinds of nonlinearities simultaneously exist. 

**Figure 7:**
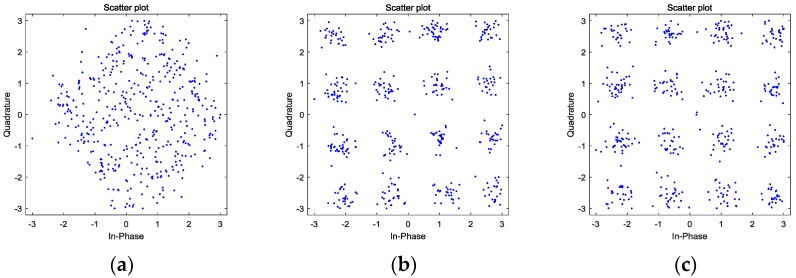
The constellations of the 16QAM signals in the polluted regions: (**a**) distorted weak 16QAM; (**b**) weak 16QAM after mitigation without delay; and (**c**) weak 16QAM after mitigation at the best delay.

Figure 7 shows the constellations of the weak 16QAM in Figure 5 before and after mitigation. Due to the nonlinear components coming from the spectrum hyperplasia of adjacent strong 16QAM, the weak signal is interfered and its SINR is not large enough to directly carry on demodulation, thus its constellation is randomly distributed in Figure 5a. Meanwhile, in Figure 5b, benefiting from the mitigation method, the intermodulation in the polluted region is obviously depressed, finally obtaining a regularly-distributed constellation. This experiment illustrates that the proposed nonlinear distortion mitigation method can improve the ability of detecting weak signals, which is a crucial capacity of sensing performance.

Since the constellations in Figure 7b,c seem to be nearly the same, their error vector magnitudes (EVMs) and bit error rates (BERs) are calculated to specifically explain the advantage of the best delay-based nonlinearity mitigation method. After normalization, the result is obtained and shown in Table 1. Compared to the results without the best delay, when the best delay condition is met, the EVM is lower and the BER is also better, as expected. As mentioned above, the major components distorting the weak 16QAM belong to the first kind of intermodulation product. Since both the mitigation methods can effectively eliminate these nonlinearities and result in a considerable SFDR increase after nonlinearity elimination, it is rational that the EVM and BER achieved by the best delay-based approach do not have an additional increase in the order of magnitude.

### 4.2. The Verification of Signals from a Real-World RF Device 

In this part, signals passing through real-world RF are utilized for testing. For the sake of corresponding with the simulation signal experiments, the signals used for testing contain three strong 16QAM signals and one weak 16QAM signal. The sampling rate here is 112 MHz. The strong 16QAMs have the same symbol rate of 2 MHz and their center frequencies are 157 MHz, 162 MHz, and 168 MHz, respectively. The weak one, with a 0.5 MHz symbol rate, is located at 150 MHz. The center frequency of the intermediate frequency sampling is set as 160 MHz and the passband bandwidth is 40 MHz. 

**Figure 8:**
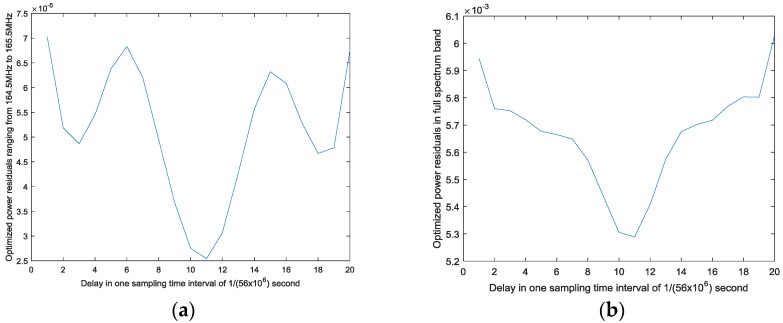
Relationships between delay and power residuals after optimization: (**a**) the optimized power residuals in the false-alarm region; and (**b**) the optimized power residuals in the full-spectrum band.

**Figure 9:**
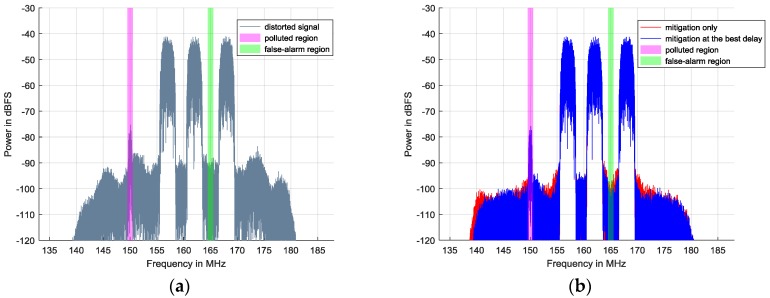
Nonlinearity mitigation results of simulation signals including three strong 16QAM signals and one weak 16QAM signal: (**a**) distorted signals; and (**b**) signals after nonlinearity mitigation.

The order and memory depth of the Hammerstein model employed is set to be the same as those of the simulation signals. From Figure 8b, among the optimized power residuals of each delay, when delay τ equals 11, the minimal value is 88.3% of the optimized E(ω,τ) without the best delay. This indicates that nearly 11.7% of power residuals, when mitigation is already finished without the best delay, can still be reduced if best delay searching is carried out. In Figure 8a, the best delay is also decided as 11, which reduces the optimized power residuals in the false-alarm region to almost one third of that without delay. A comparison of the spectrum before and after nonlinearity compensation is shown in Figure 9. As expected, SFDR increases at least 10 dB and the best delay approach can eliminate 3–5 dB more nonlinearities in most parts of the spectrum.

To verify the improvement of spectrum sensing performance of the proposed mitigation method, the green region in Figure 9 is chosen as the band of interest. Its receiver operating characteristics (ROC) for each case are calculated for the detection of spurious signals. The ED [37] method is utilized as a demonstration where the detection threshold is calculated by:

(36) $$\gamma =\sigma_{w}^{2}\left(N+\sqrt{2N}Q^{-1}(P_{f})\right)$$

where $\sigma_{w}^{2}$ is the noise variance of the sub-band, $Q^{-1}(\cdot )$ is the inverse function of the normal distribution function, N is the point used, and $P_{f}$ is the false-alarm probability which is used as a metric for CR-WSN failing to detect the absence of the primary signal.

**Figure 10:**
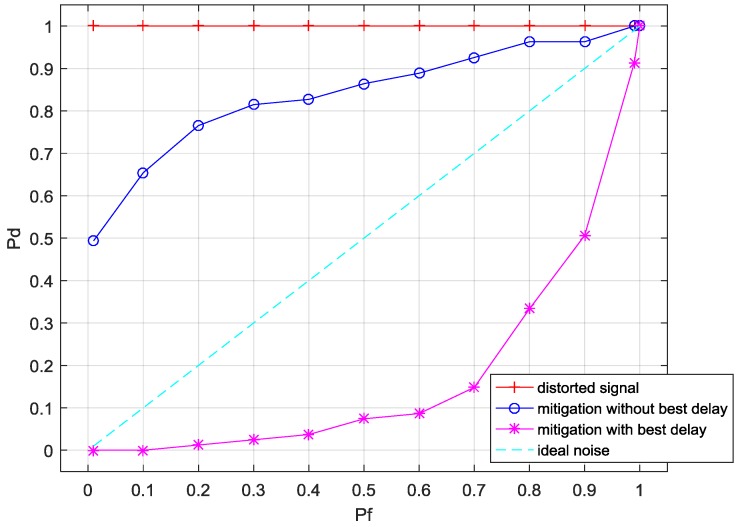
The receiver operating characteristics (ROC) before and after nonlinearity mitigation.

As shown in Figure 10, $P_{d}$ denotes the spurious signal detection probability. The curve in red represents the ROC of the distorted signal whose $P_{d}$ is always one when $P_{f}$ changes from 0 to 1. This means that, as a result of the existence of intermodulation, the ED method will always detect the green region as busy. This result is acceptable since the intermodulation component’s spectrum power is 10 dB higher than the noise floor in Figure 9a.

As for the comparison between the mitigation method with and without best delay searching, the pure noise’s ROC is treated as a standard whose characteristic conforms to $P_{d}\approx P_{f}$ . Considering that the ROC curve here is in the false-alarm region, $P_{d}$ indicates the probability of detecting a nonexistent signal. For a fixed probability of $P_{f}$ , the blue curve’s $P_{d}$ is always greater than that of the pure noise, while the purple’s curve is always less. This is to say that, by the proposed best delay-based mitigation method, the sensor is less likely to detect a false signal. Consequently, we can draw the conclusion that nonlinearities can be mitigated more effectively when best delay searching is employed and, in that way, the reliability of spectrum sensing is enhanced.

**Figure 11:**
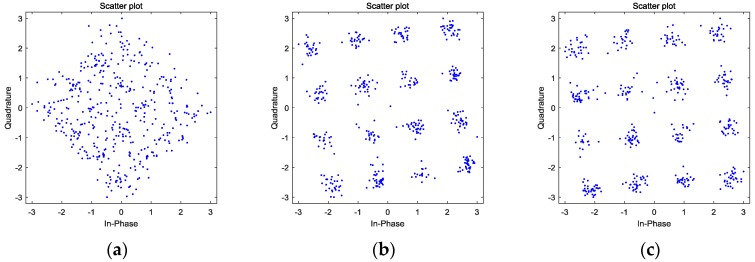
The constellations of the 16QAM signals in the polluted regions: (**a**) distorted weak 16QAM; (**b**) weak 16QAM after mitigation without delay; and (**c**) weak 16QAM after mitigation at the best delay.

Finally, constellations of the distorted weak 16QAM by the intermodulation components are calculated in Figure 11. Randomly-distributed constellation points become regular after the nonlinearities are eliminated. This result is also the same as that obtained in the simulation signal. Therefore, when handling real-world signals, the proposed nonlinearity mitigation method achieves the desired result.

To sum up, the performance of the proposed method is demonstrated through the results of the simulation signals and signals from real-world measurements. The suppression of the nonlinear components is significantly better when best delay searching is performed. Thus, the proposed method provides a high-performance linearization solution for a broadband RF front-end in a sensor node.

## 5. Conclusions

In this paper, aiming to compensate for the intermodulation components brought by adjacent strong signals, we offer the best delay searching-based nonlinearity mitigation method to enhance the SFDR of the RF front-end and improve the reliability of spectrum sensing in CR-WSN. To summarize, the advantages of the proposed method are as follows:

1. There are plenty of non-ideal linear characteristic modules bringing in nonlinear distortions in the RF front-end of a sensor. Moreover, when the group delay distortion coming from the time delay electronic components joins with nonlinearities, it is necessary to design this blind identification criterion of nonlinearity mitigation at the best delay. Here, the best delay means the sampling interval between the output of the nonlinear behavior model and the distorted signal which corresponds to the minimal power residual after optimization.
2. Based on the proposed identification criterion, the mitigation architecture with best delay searching is presented. In this architecture, an odd-order generalized Hammerstein model is utilized as the nonlinear behavior model to compensate for the intermodulation components of the strong signals. Additionally, a flexible band-split method with low complexity is provided in the frequency domain to save both amplitudes and phases contained in the original signals. Finally, to solve the system of linear equations in the complex field which, in most cases, is ill-conditioned, the WII method is employed.

In conclusion, the proposed method achieves satisfactory results when tested both on the simulation signals and signals from real-world measurements. If applying it to the RF front-end in the sensor, the performance of spectrum sensing can be improved predictably. Our next stage is to find a better solution for modeling the group delay distortion instead of using only sampling interval changes. This further development is beyond of the main scope of this paper, but offers an interesting and relevant topic for future studies.

## Figures and Tables

**Table 1:** EVM and BER of weak 16QAM after nonlinearity mitigation with or without the best delay.

	Mitigation without the Best Delay	Mitigation with the Best Delay
EVM	22.3%	15.1%
BER	8.54 × 10^−3^	3.12 × 10^−3^

## Abbreviations

The following abbreviations are used in this manuscript:

CR-WSN	Cognitive Radio Wireless Sensor Network
SFDR RF	Spurious-Free Dynamic Range Radio Frequency
WSN	Wireless Sensor Network
MEMS	Micro-Electro-Mechanical Systems
ISM	Industrial Scientific Medical
CR	Cognitive Radio
HCRSN	Heterogeneous Cognitive Radio Sensor Network
SU	Secondary User
PU	Primary User
I/Q	In-Phase/Quadrature
DCR	Direct-Conversion Receiver
ADC	Analog-to-Digital Converter
DDC	Digital Down-Conversion
DSP	Digital Signal Processing
FIR	Impulse Response Filter
CDF	Cumulative Distribution Function
LNA	Low Noise Amplifier
SNR	Signal Noise Ratio
IFFT	Inverse Fast Fourier Transform
WII	Weighed Iterative Improvement method
IF	Intermediate Frequency
BPF	Band-Pass Filter
DFT IDFT	Discrete Fourier Transform Inverse Discrete Fourier Transform
FFT	Fast Fourier Transform
RLS	Recursive Least Squares method
SDR	Software Defined Radio
ED	Energy Detection
SINR	Signal to Interference plus Noise Ratio
EVM	Error Vector Magnitude
BER	Bit Error Rate
ROC	Receiver Operating Characteristics
